# Bibliographical

**Published:** 1856-07

**Authors:** 


### BIBLIOGRAPHICAL.

*On Bandaging and other Operations of Minor Surgery.* By F. W. Sargent, M. D., Member of the College of Physicians of Will's Hospital, etc., etc. New edition revised and enlarged, with One Hundred and Eighty-One Illustrations, Philadelphia; Blanchard & Lea, 1856.

This is a most excellent little work, and should be in the hands of every student and young practitioner. In the more complete treatises on surgery, and also in the lecture room, various minor operations and appliances that are of vast importance both to the surgeon and practitioner, are only cursorily noticed or entirely omitted; the young practitioner commencing the practice of his profession, with a very imperfect idea of this important part of practical medicine. This work most admirably fills this deficiency. The work has reached its second edition. The illustrations are well executed, and are perfectly intelligible to the youngest students.

For the contents we extract the following from the preface:

"The book is divided into five parts. Of these, the first embraces a description of the implements, if such a term be admissible, with which the ordinary duties of the surgeon are accomplished.

"The second treats of the composition and preparation of Bandages, of their application to the different regions of the body, and of the purposes which they are thus made to subserve.

"The third is devoted to the consideration of the apparatus of various kinds, used in the treatment of Fractures. In the arrangement of this portion of the volume, the Author has thought it expedient to give pretty full details, showing the indications of treatment in each particular case of Fracture, and thereby rendering more manifest the adaptation of each bandage, splint, or other dressing, to the fulfilment of these requirements.

"The fourth division describes the mechanical means employed in the treatment of dislocations, with the mode of applying them.

"In the fifth part are detailed at length the methods of performing such operations as seem strictly to be included in the term "Minor Surgery;" these are the operations for bleeding, general and local; the modes of effecting counter-irritation; the methods of arresting hæmorrhage; the closure of wounds; the introduction of the catheter, and the administration of injections. A few remarks on the mode of relieving pain during operations, and a short appendix of useful formulæ, close the volume."

Received through Messrs. J. J. Richards & Co., of this City.

---

*A Practical Treatise on the Diseases of the Testis, and of the Spermatic Cord and Scrotum; with numerous wood engravings.* By T. B. Curling, F. R. S., Surgeon to the London Hospital, Lecturer on Surgery at the London Hospital Medical College, President of the Hunterian Society, London, etc. Second revised and enlarged edition. Philadelphia: Blanchard & Lea, 1856.

This is certainly the most complete work upon this subject, and shows in an eminent degree the great research of the Author. It is now, both in Europe and America, considered the standard work upon diseases of the testicle and spermatic cord. It may be seen that the second edition differs in some particulars from the first, published more than twelve years ago. Upon this subject, the author says: "In this edition, some new chapters have been added; many have been re-written or altered; and, it is hoped, that nearly all of them contain additional facts of practical interest and importance. By omitting the anatomy, which formed the introductory part of the first edition, and by careful conciseness in the description of cases, I have been able to embody a great deal of fresh matter, and to add several new wood engravings without enlargement of the volume."

The statistical record of the various diseases, and their treatment comprise an interesting feature of the work, and to show how voluminous, we will make one or two extracts in regard to the statistics of Hydrocele. In speaking of the age at which it most frequently occurs, he says: "In a table of 1000 cases of hydrocele treated by iodine injection at the Native Hospital of Calcutta, it appears that none of the patients operated on were less than eighteen years of age; about one twenty-fourth were not more than twenty years old; rather more than a sixteenth were from twenty-one to twenty-five years of age; a little less than half from twenty-eight to thirty-five; a little more than a quarter from thirty-six to forty-five; and an eighteenth were upwards of forty-six years."

And in speaking of the treatment by the injection of iodine, says: "Iodine injections were first employed by Mr. Martin, formerly a surgeon in India. He used the tincture in the proportion of ʒij—ʒvj of water; injected only a small quantity; and instead of afterwards withdrawing the fluid, allowed it to remain in the sac to be removed by absorption. In a report of cases of hydrocele thus treated at the Native Hospital of Calcutta, it is stated that from the 9th of March, 1832, to 31st of December, 1839, 2393 cases were under treatment. Of these were

| | |
|---|---|
| Hindus,.............................................. | 1265 |
| Mahometans,...................................... | 1076 |
| Christians,.......................................... | 52 |
| Total,.............................................. | 2393 |

And it appears that the failures were rather under one per cent.; a result which must be regarded as remarkably successful. Within the last ten years these injections have been extensively tried in Europe, and with a success which has led to their pretty general use in this country. I do not believe, as some have supposed, that iodine exerts any peculiar or specific influence on the serous sac. Like other injections it acts as a stimulant, stirring up inflammation, and like them also, it is liable occasionally to fail in persons insusceptible to inflammatory excitement, though the retention of a portion of the injection in the sac more certainly insures a favorable result."

It is certainly an invaluable work to all who wish to become proficient in this class of Diseases. Received through the hands of Messrs. J. J. Richards & Co., of this City.

